# Rearing and observation of immature stages of the hoverfly *Microdon katsurai* (Diptera, Syrphidae)

**DOI:** 10.3897/BDJ.4.e10185

**Published:** 2016-12-09

**Authors:** Hironori Iwai, Daiki D Horikawa, Kazuharu Arakawa, Masaru Tomita, Takashi Komatsu, Munetoshi Maruyama

**Affiliations:** ‡ Institute for Advanced Biosciences, Keio University, Tsuruoka, Japan; § Faculty of Environmental and Information Studies, Keio University, Fujisawa, Japan; | Systems Biology Program, Graduate School of Media and Governance, Keio University, Fujisawa, Japan; ¶ The Kyushu University Museum, Fukuoka, Japan

**Keywords:** Hover fly, Microdontinae, host record, *Polyrhachis lamellidens*, myrmecophily, puparium

## Abstract

**Background:**

The hoverfly Microdon (Chymophila) katsurai Maruyama et Hironaga 2004 was speculated to be a myrmecophilous species associated with the ant *Polyrhachis lamellidens* based on observations of adults near the ant nest. However, there have been no reports regarding the observation of immature stages of this species in association with *P. lamellidens*.

**New information:**

For the first time, we found three *M. katsurai* larvae inside a *P. lamellidens* nest and conducted rearing experiments on the larval *M. katsurai*. *P. lamellidens* workers did not show any inspection or attack behavior against the *M. katsurai* larvae under rearing conditions, suggesting that *M. katsurai* larvae can survive inside a *P. lamellidens* nest. Although no predatory behavior by the *M. katsurai* larvae was observed, all the *M. katsurai* larvae pupated and emerged in a rearing environment. The dorsal surface of the larval *M. katsurai* has a distinct pale green color with a uniform reticular structure. The puparium of *M. katsurai* shows several morphological features that are characteristic of the subgenus *Chymophila*. We conclude that *M. katsurai* is likely a myrmecophilous species that utilizes *P. lamellidens* as a specific host and that classification of *M. katsurai* based on puparium morphology is concordant with that based on adult morphology.

## Introduction

It is well known that some species of Microdontinae (Diptera, Syrphidae) spend their larval and pupal periods in ant nests. Several papers reported that larvae of some microdontine species prey on ant eggs, larvae, and pupae (Van Pelt and Van Pelt 1972, Duffield 1981, Garnett et al. 1985, Reemer 2013, Pérez-Lachaud et al. 2014). At present, 454 microdontine species have been recorded (Reemer and Stahls 2013), and host species for 49 of them have been confirmed as myrmecophilous (Maruyama et al. 2013, Reemer 2013). However, there are few studies on the biology of myrmecophilous microdontine species in Asia (Maruyama and Hironaga 2004).

The microdontine species *Microdon katsurai* was originally described from Japan (Maruyama and Hironaga 2004). Adult *M. katsurai* have a remarkable greenish yellow luster. Adult stage *M. katsurai* are found near nests of the ant *Polyrhachis lamellidens* Smith 1874 (Hymenoptera, Formicidae) from the end of May to the beginning of July (Hironaga et al. 1998, Katsura 1998, Maruyama and Hironaga 2004). Furthermore, adult females of *M. katsurai* show ovipositional behavior at the entrance of *P. lamellidens* nests (Hironaga et al. 1998, Maruyama and Hironaga 2004). These studies imply that *P. lamellidens* is a host of *M. katsurai*. However, *M. katsurai* has never been found inside a *P. lamellidens* nest, and no interactions between these two species have been observed.

In the present study, to elucidate whether *M. katsurai* utilizes *P. lamellidens*, we tried to collect larvae of *M. katsurai* from a *P. lamellidens* nest and rear them until they developed into adults.

## Materials and Methods

### Collecting samples

**Figure 1.**
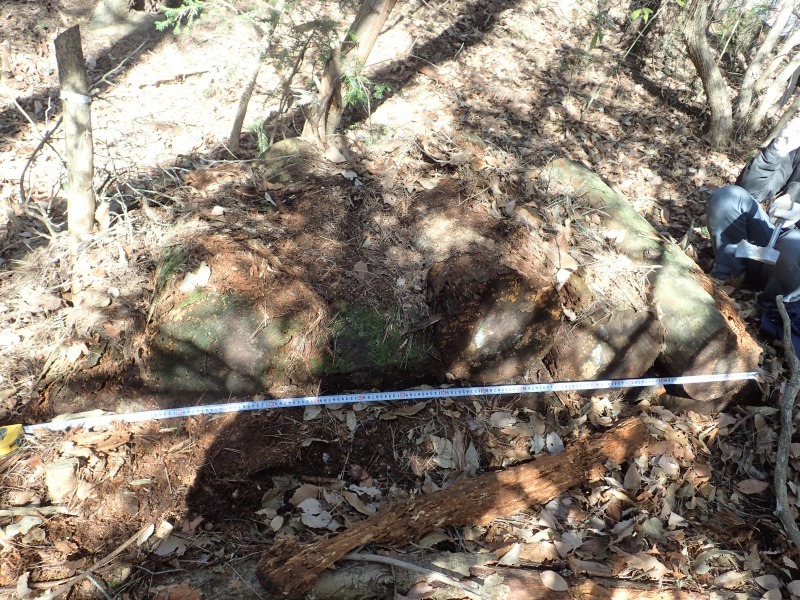
The nest of *Polyrhachis lamellidens* in which microdontine larvae and puparia were found.

We conducted field research on 5 March, 6 March, 15 March, and 16 March 2015 in Hosaka Natural Park (N 35°43'55", E 138°29'18", approximately 600 m in altitude), in the city of Nirasaki, Yamanashi Prefecture, Japan. We found a *P. lamellidens* nest in several rotten *Pinus densiflora* Siebold et Zuccarini 1842 trees that had fallen and were piled up (Fig. 1). Each tree was approximately 100 cm in length and 20 to 50 cm in diameter.

**Figure 2.**
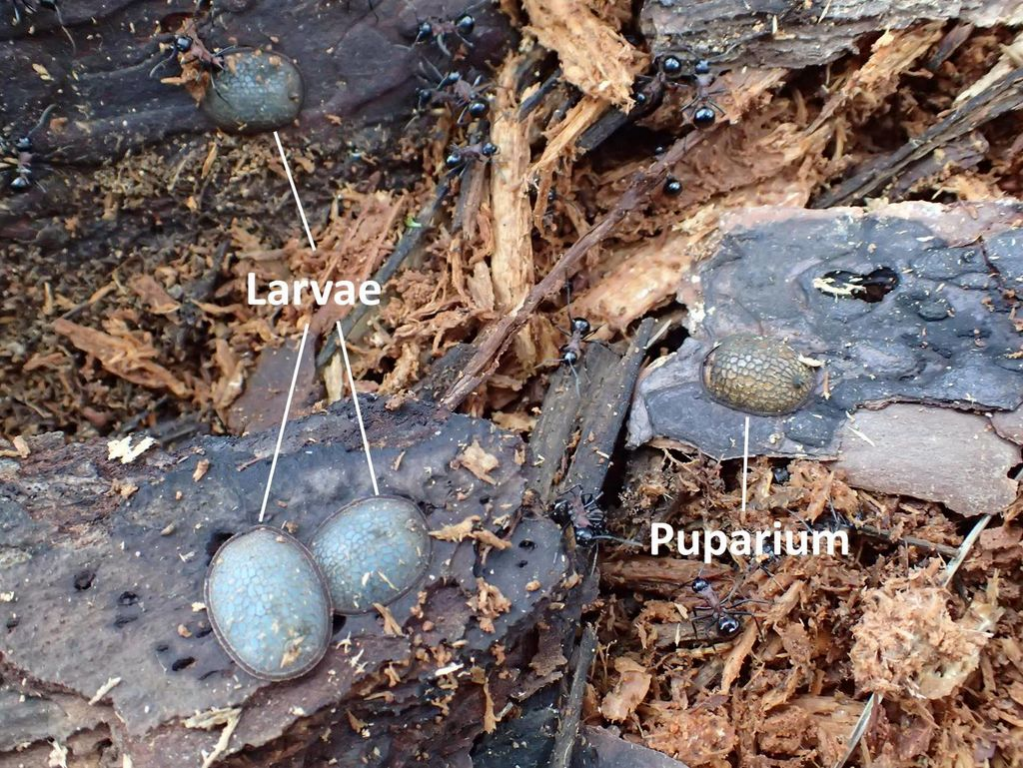
The microdontine larvae and puparia discovered in the *P. lamellidens* nest. Three larvae and one puparium were attached to pieces of bark.

**Figure 3.**
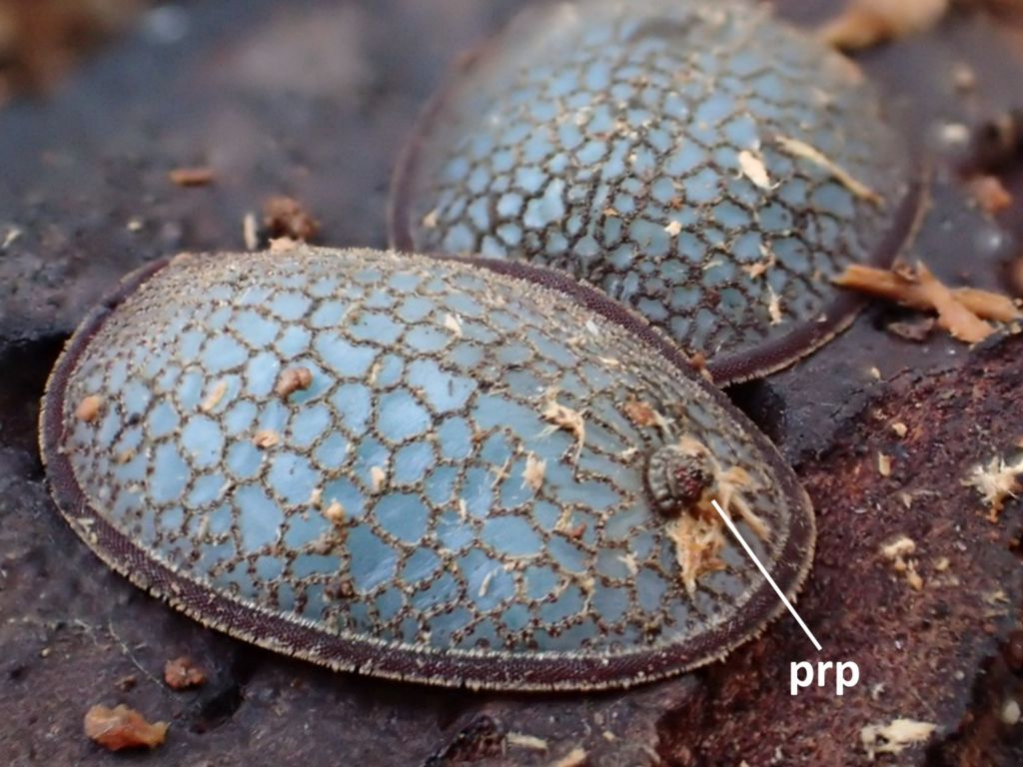
A posterior respiratory process (prp) was observed on a *Microdon katsurai* larva.

**Figure 4.**
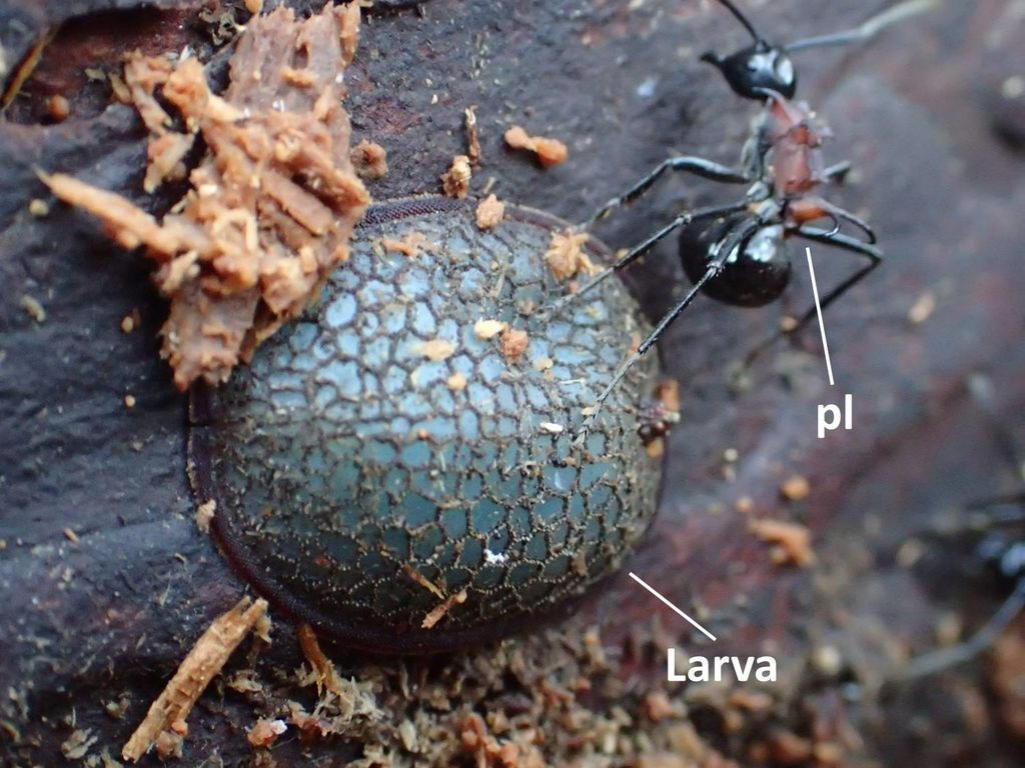
A *P. lamellidens* worker passing by a *M. katsurai* larva; pl refers to *P. lamellidens* worker.

**Figure 5.**
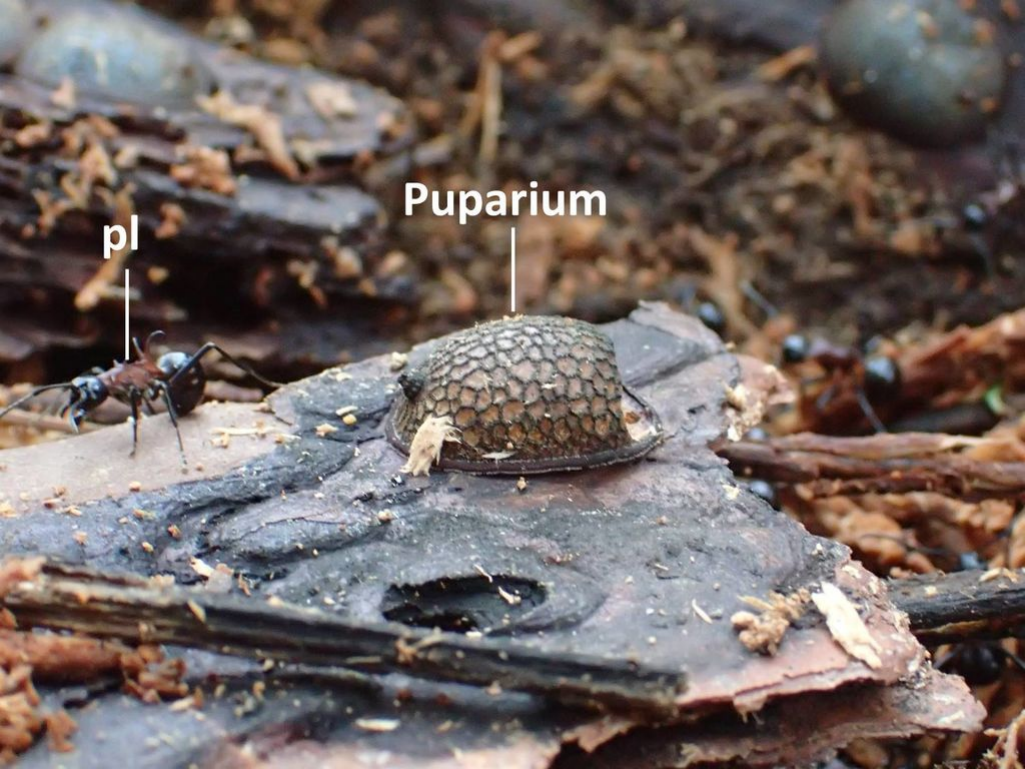
Puparium of *M. katsurai* with a *P. lamellidens* worker; pl refers to *P. lamellidens* worker.

Three larvae and four puparia of a microdontine were found in the *P. lamellidens* nest (Figs 2, 3, 4, 5). These larvae were found on a piece of bark in the underground nest of *P. lamellidens*. Two of the puparia were attached onto a piece of the bark as well, and the other two were found in wood flakes in the *P. lamellidens* nest. The larva with the pieces of bark were taken and transferred into a transparent plastic container in which wood flakes derived from the *P. lamellidens* nest were placed on the bottom. All workers and queens of *P. lamellidens* found in the nest were collected and transferred into a plastic container. All the collected samples were brought to the laboratory.

### Rearing animals

Due to the difficulty of identifying microdontine species by using a larva or pupa, we reared the larvae collected until adults emerged under laboratory conditions. After collecting these larvae, they were reared in the transparent plastic container used for collecting the larval samples for the first 2 d. Thereafter, the larvae were numbered 1 to 3 and reared individually in a new plastic container (130 mm in diameter and 59 mm in height), and wood flakes taken from the *P. lamellidens* nest were placed on the bottom. Small holes were made in the lid of each container for ventilation. At 2 d after the rearing experiments began on 15 March 2015, several *P. lamellidens* larvae brought from their nest were introduced to each container as a potential food source for the microdontine larvae. Additionally, 80 adult workers were introduced to each container 5 d after rearing started. Furthermore, maple syrup diluted in water at a ratio of 1:1 and placed on a small piece of aluminium foil was supplied in each rearing container every 3 d.

Each rearing container was kept at approximately 25ºC in a darkroom. Observations of the animals were conducted at intervals of 1-3 d until they became adults. During this period, interaction behaviors between the microdontine larvae and the ants were recorded as well. Additionally, to better observe the ventral morphology of the larva, we detached one larva (No. 2) from a piece of bark flake and placed it to the side wall of the transparent container. The ventral surface of the larva is sticky enough to attach onto the container wall. *P. lamellidens* workers were removed before the microdontine adults emerged to avoid any attack behavior of ant workers against adult hover flies (Wheeler 1908, Akre et al. 1973).

### Morphological studies

We conducted observations on the morphology of the microdontines at the larval and adult stages. The body color and morphology of the dorsal surface were recorded in individual larvae, prepupae (this stage lies between the moment when the cuticle of the larva hardens and the appearance of the anterior spiracles (Akre et al. 1973, Akre et al. 1988)) and pupae. Morphological observations of the puparium were conducted using all the individuals obtained, whereas only one individual emerged successfully and was used for morphological observation of the adult. The adult was prepared as a dry specimen after killing at -20ºC. The sample was kept in the freezer until subsequent morphological observation for species identification was conducted. The puparium was detached from the surface of the piece of bark using invert soap. Morphological observations were conducted according to Maruyama and Hironaga (2004) for the adult and Thompson (1981), Rotheray 1993 and Oishi (1996) for the puparium. The anterior spiracles and posterior respiratory process of the puparium, and dorsal views of the adult and puparium were photographed with a Canon Eos D60 with an MP-E65 macrolens several times with differing focus. These photographs were then synthesized by depth synthetic processing using the Combine ZP software (Hadley 2010). The length, width, and height of the puparium were measured using a vernier caliper. Furthermore, height and width of the both anterior spiracles and a marginal band were measured using the VHX-5000 system (Keyence).

## Results

### Development of a microdontine species under rearing conditions

The microdontine larvae did not show any predatory behavior against *P. lamellidens* larvae throughout the rearing experiments. Neither was any attack behavior of *P. lamellidens* workers against the microdontine larvae and pupae observed. One of the larvae (No. 2) started pupation 24 d after collection in the field, and the other two (No. 1 and 3) began pupation 16 d after that (Table 1). Since no exuviae were found in the container prior to pupation of the larvae, the larvae were at the final instar stage at the time they were collected in the field. A pair of discs was observed on the dorsal front part of the individuals (Fig. 7). The appearance of a pair of anterior spiracles from the discs, which is a signature of development from prepupa to pupa, was confirmed 3 d after the beginning of pupation in individual No. 2 and 8 d after the beginning of pupation in No. 1 and 3.

Individuals No. 1 and 3 emerged as adults 25 d after the beginning of pupation (Table 1, Fig. 9). Both individuals failed to expand their wings, presumably because of a lack of an appropriate substrate for the individuals to mount on and expand their wings appropriately. These two died 2 d after their emergence.

We set a ladder-like scaffold made of a egg paper tray inside a corner of the container for individual No. 2 to mount on 17 d after pupation. In addition, the lid was removed from the container since we noticed that there was condensed water due to the high relative humidity on the inner surface of the container. Then, individual No. 2 emerged with fully expanded wings. The adult of individual No. 2 was transferred into a small glass vial and killed by subjecting it to a temperature of -20ºC in a freezer 6 d after emergence.

### Morphology of larval, prepupal, pupal, puparial, and adult Microdontinae

**Figure 6.**
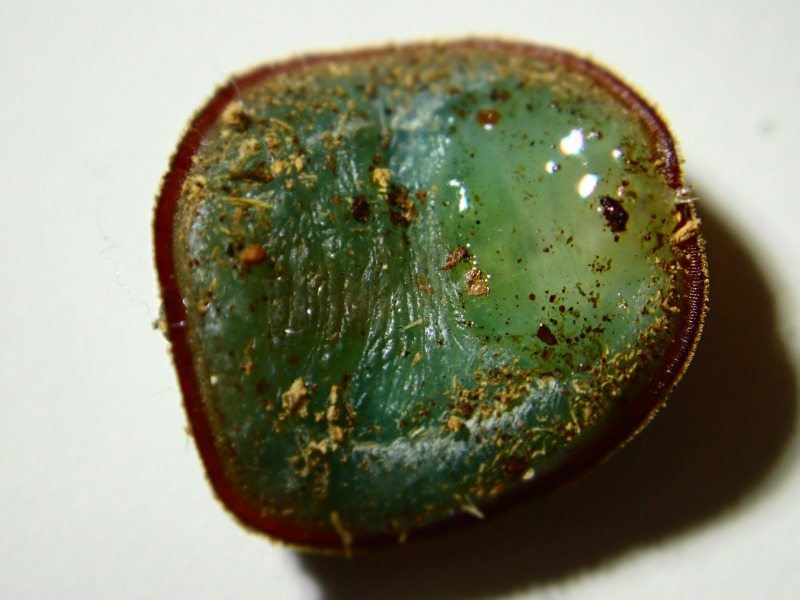
Ventral view of the *M. katsurai* larva (individual No. 2).

**Figure 7.**
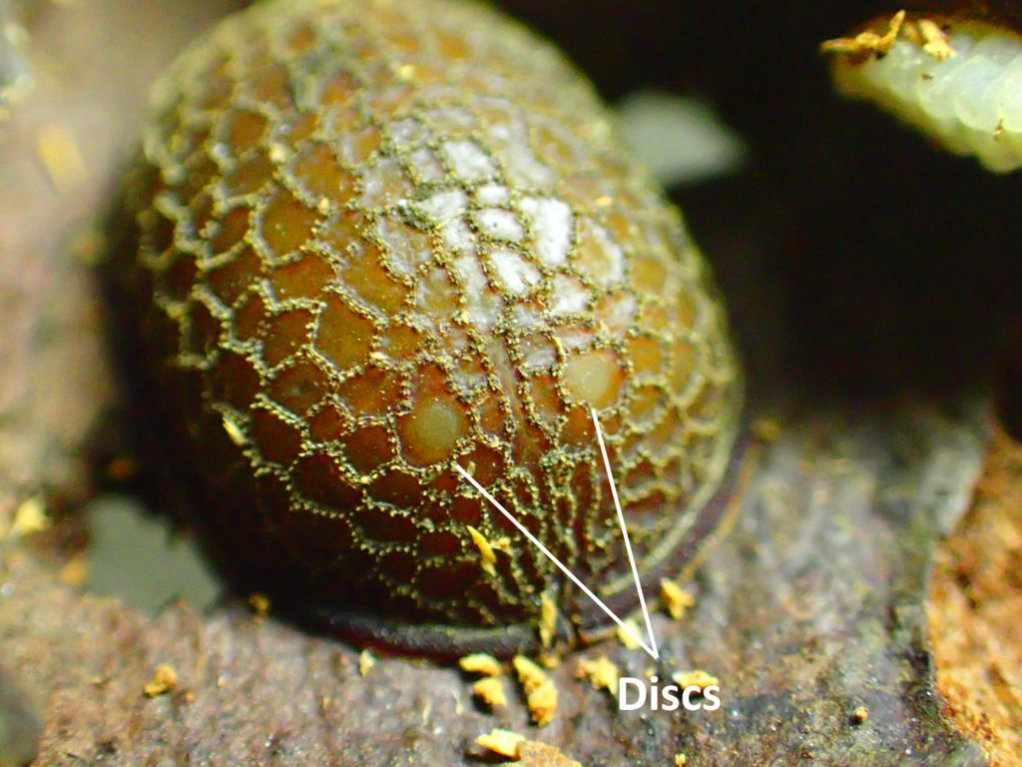
A pair of discs on the dorsal front part of the prepupa.

**Figure 8.**
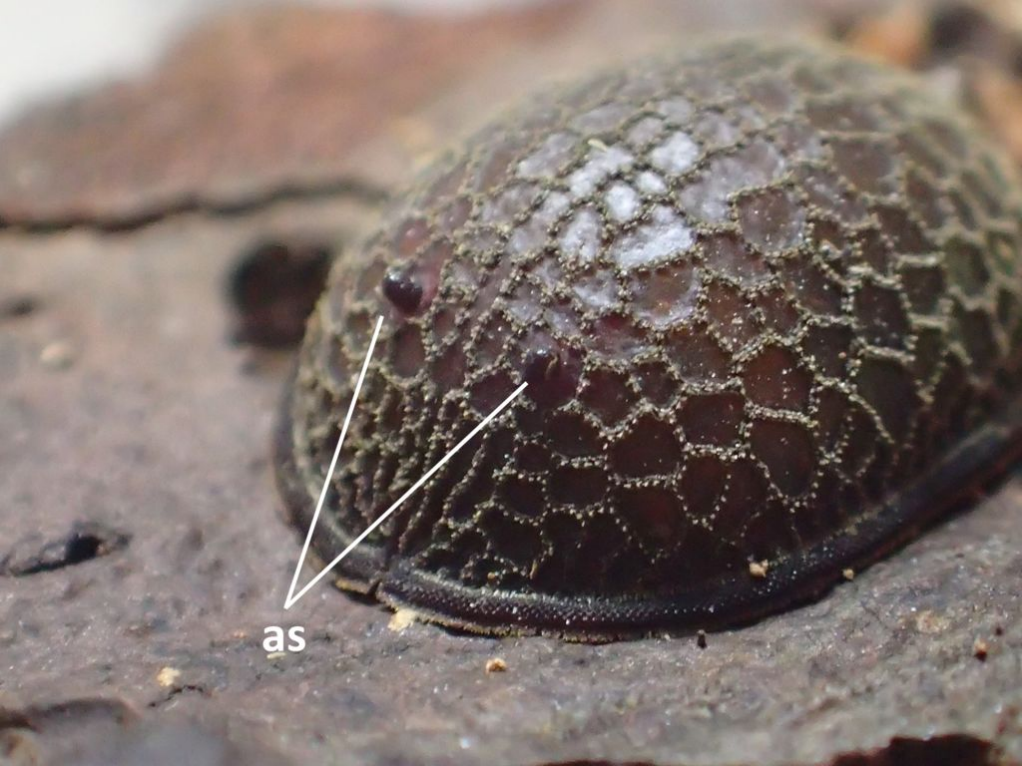
A pair of anterior spiracles (as) protruded from the discs.

**Figure 9.**
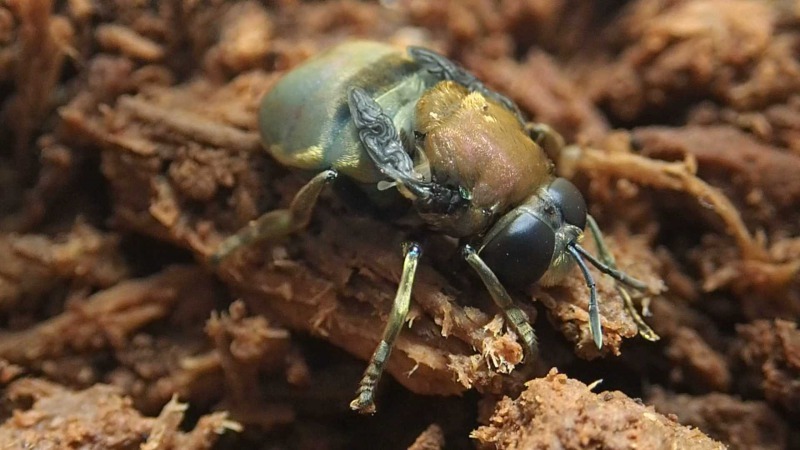
A microdontine adult (individual No. 3) with eclosion insufficiency. It has unexpanded wings.

Morphological observations of the microdontine species were conducted at different developmental stages. All the microdontine larvae had a dark brown reticulated structure with a pale green color on the overall dorsal surface (Figs 3, 4). The ventral surface of the larva had an emerald green color (Fig. 6). The whole-body color of the larva changed to light brown when it entered the prepupa stage (Fig. 7). A uniform reticular structure was observed on the dorsal surface of the prepupae and pupae as well as the larvae (Figs 7, 8). The body color of the pupa became dark brown 3 to 8 d after the beginning of pupation (Fig. 8).

**Figure 10.**
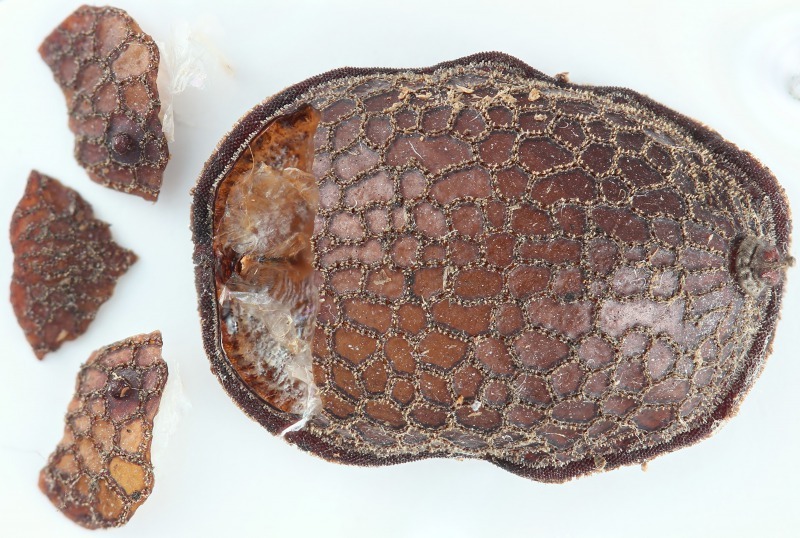
A microdontine puparium, dorsal view (individual No. 2). Pieces of the puparium (left) formed after eclosion.

**Figure 11.**
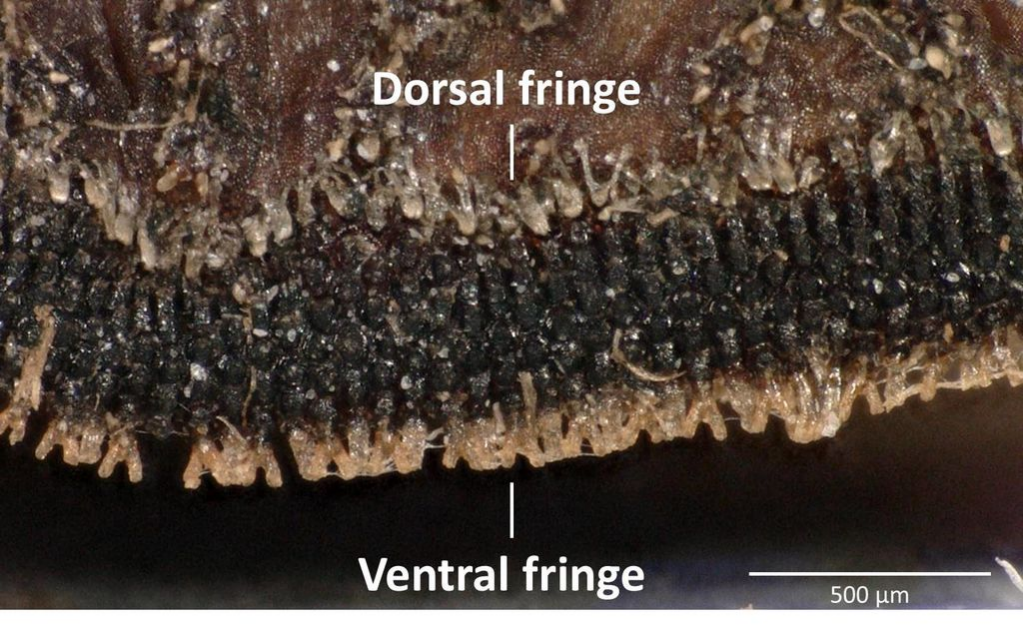
Lateral view of the puparium edge (individual No. 2). A dorsal fringe and ventral fringe were observed.

**Figure 12.**
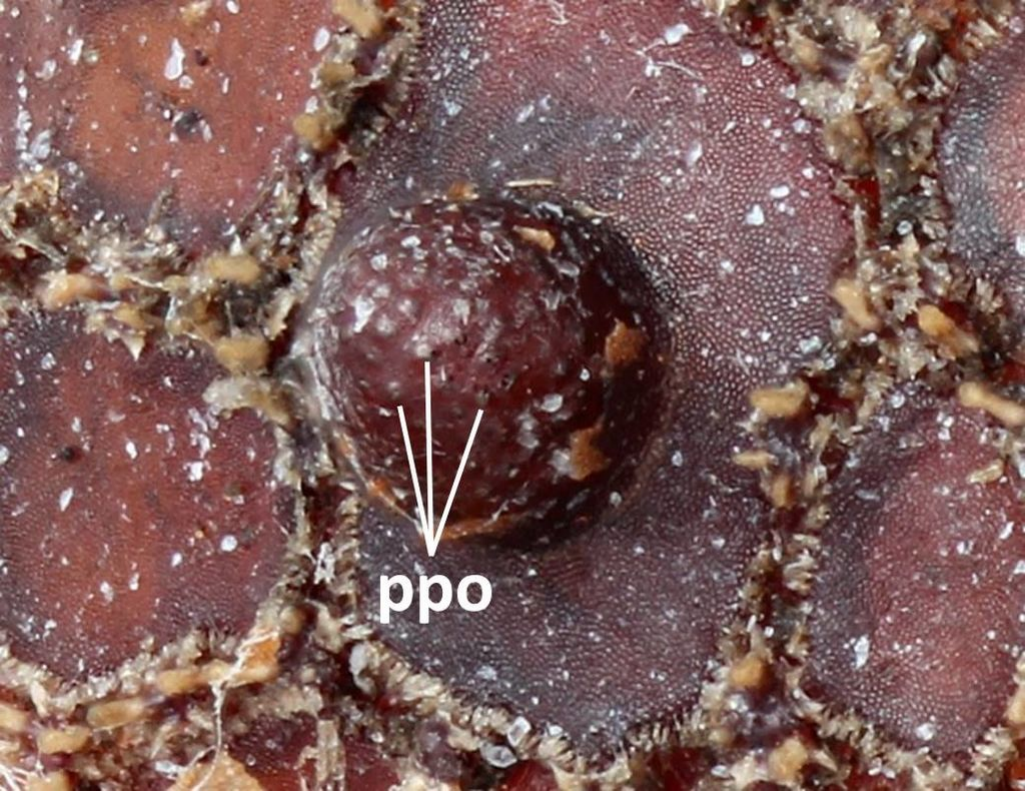
Anterior spiracle, lateral view (individual No. 2). ppo refers to protruded pores.

**Figure 13.**
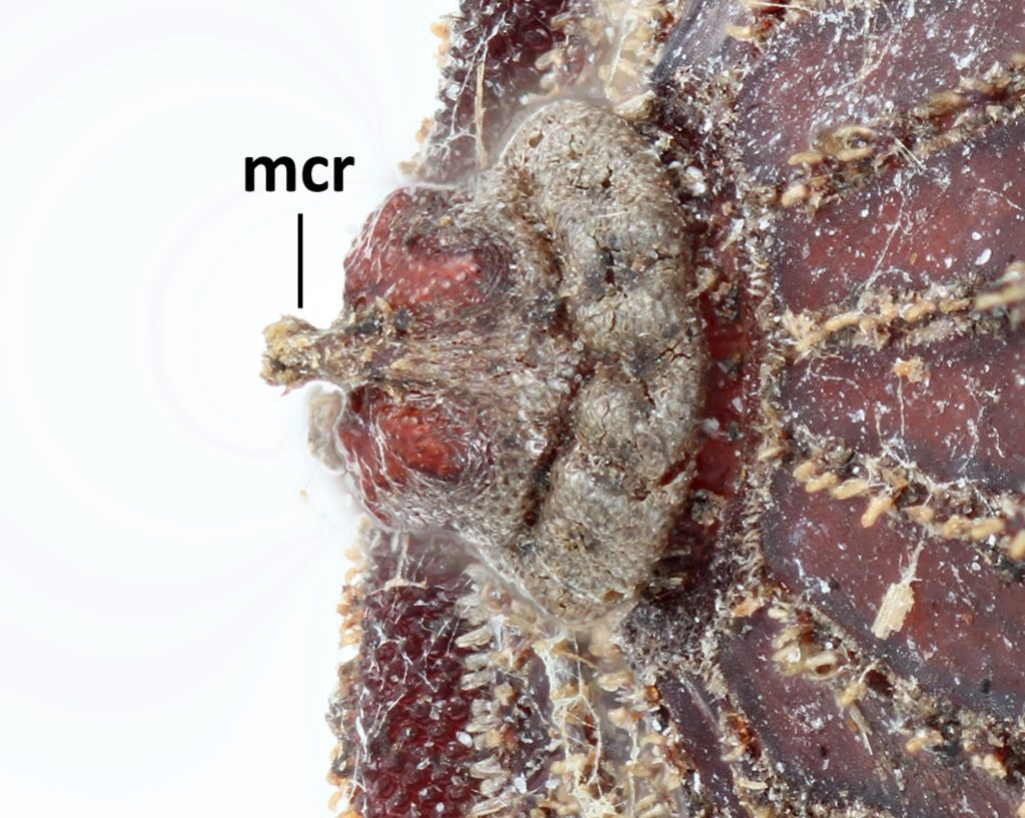
Posterior respiratory process, dorsal view (individual No. 2); mcr refers to median carina.

**Figure 14.**
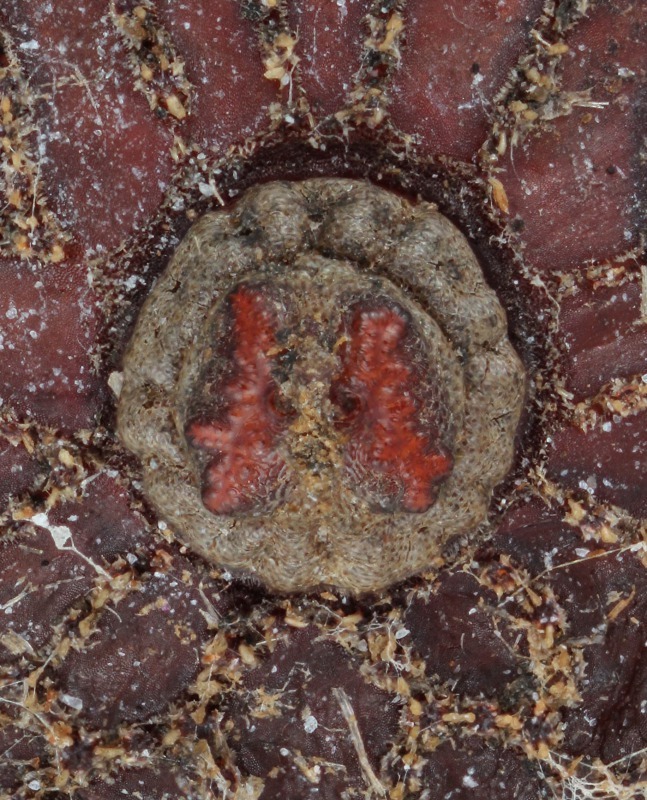
Posterior respiratory process, caudal view (individual No. 2).

Three pieces were made from the puparium shown on the left side in Fig. 10 when the adult emerged. These puparia were 12.8-13.1 mm in length (*N* = 3), 8.6-8.8 mm in width (*N* = 3) and 6.0 mm in height (*N* = 1: the heights of puparia No. 1 and No. 3 were not measured because the dorsal surfaces of these puparia were damaged). The thick marginal band consisted of both dorsal fringe and ventral fringe (Fig. 11). The anterior spiracle on the right side was 398-450 µm in height and 464-488 μm in width (*N* = 2: the right anterior spiracle of No. 1 was not measured because it was lost at the time emergence) and that on the left side was 410-465 μm in height and 484-497 μm in width (*N* = 3). In addition, protruding pores were observed on the surface of the anterior spiracles in each individual (Fig. 12). The spiracular opening at the posterior respiratory process was separated into two areas by a flat median carina (Figs 13, 14).

**Figure 15.**
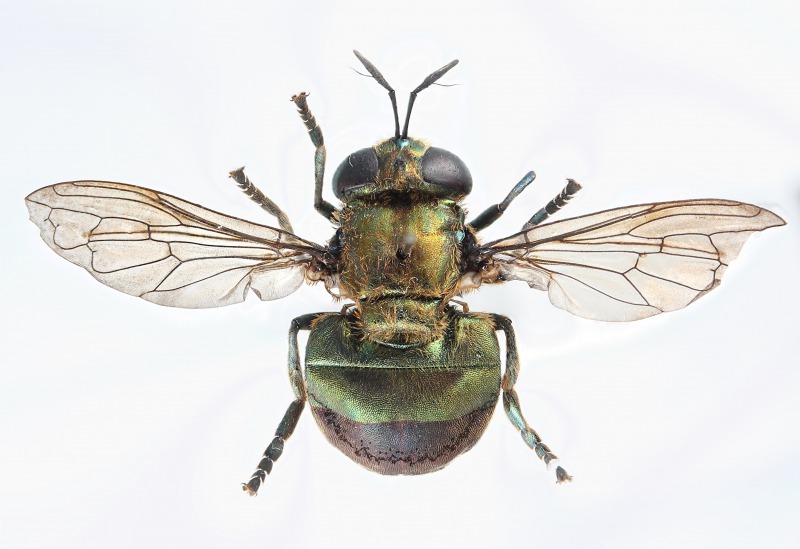
Dorsal view of the microdontine adult (individual No. 2).

Concerning the adult, observation was conducted on the specimen casted by individual No. 2 only, which successfully emerged (Fig. 15). The adult of individual No. 2 had a brilliant pale green color with metallic luster except for the caudal part of the abdomen, which was dark purple (Fig. 15). This adult specimen was identified as a female of *Microdon katsurai* according to Maruyama and Hironaga (2004).

## Discussion

Based on observations of the appearance and ovipositional behavior of adult *M. katsurai* near *P. lamellidens* nests (Hironaga et al. 1998, Katsura 1998, Maruyama and Hironaga 2004), *M. katsurai* was speculated to spend its immature stages in *P. lamellidens* nests. In the present study, for the first time, we found *M. katsurai* larvae from the inside of a *P. lamellidens* nest and succeeded in rearing them to the adult stage when they were reared with *P. lamellidens* workers and larvae. This finding provides firm evidence that *M. katsurai* utilizes *P. lamellidens* nests. *M. katsurai* has never been found near nests of other ant species, suggesting that it is a host-specific myrmecophilous species.

*P. lamellidens* workers were not observed to inspect or attack the *M. katsurai* larvae in the rearing environment, suggesting that *M. katsurai* larvae can survive inside a *P. lamellidens* nest. Howard et al. (1990) demonstrated that the cuticular chemical profiles of the larvae of the myrmecophilous Microdontinae species *Microdon albicomatus* Novak 1977 is the same as that of its prey, the pupae of the ant *Myrmica incompleta* Provancher 1881. *M. katsurai* larvae might also employ such chemical mimicry for avoidance of attacks by *P. lamellidens*, although no data on cuticular chemical profiles in this species are available. We cannot conclude that the *M. katsurai* larvae grew by preying on *P. lamellidens* because they were not observed to prey on or attack *P. lamellidens* adults or larvae during the rearing experiments. Nevertheless, all the *M. katsurai* larvae pupated and emerged (Table 1). Thus, it is obvious that these larvae had fed on some food source sufficient for them to grow into adults before we had collected them. It has been reported that larval microdontines prey on ant eggs, larvae and pupae (Van Pelt and Van Pelt 1972, Duffield 1981, Garnett et al. 1985, Reemer 2013, Pérez-Lachaud et al. 2014). Others have suggested that the larvae of certain Microdontinae feed on refuse or pellets of food ejected by ants (e.g. Wheeler 1908), but such feeding behavior has never been confirmed (Reemer 2013). Further studies in both the field and laboratory are required to reveal the types of food resources *M. katsurai* larvae utilize.

Having been collected in March, the stage of all the larvae was likely the last instar because they did not molt before becoming pupae. Adult *M. katsurai* appear from the end of May until the beginning of July (Hironaga et al. 1998, Katsura 1998, Maruyama and Hironaga 2004), suggesting that this species overwinters in an ant nest before pupation. The three individuals studied emerged between 25 April and 2 May 2015 under laboratory rearing conditions. The time of emergence in the present study was almost one month earlier than the recorded observations for adults of this species in the field (Maruyama and Hironaga 2004). This difference in appearance time may be due to the rearing temperature (25ºC) in the present study, which is higher than that of the natural habitat of this species.

The color of larvae of microdontine species is white or brown (Wheeler 1908, Wheeler 1924, Greene 1955, Dixon 1960, Rotheray 1991, Maruyama et al. 2013, Schmid et al. 2014). The pale green color shown on the dorsal surface of the *M. katsurai* larvae in this study is rare in Microdontinae; the larvae of most species in this subfamily show a white or brown color (Wheeler 1908, Wheeler 1924, Greene 1955, Dixon 1960, Rotheray 1991, Maruyama et al. 2013, Schmid et al. 2014). This is the first record in Asia of a microdontine larva with such a remarkable color.

Based on morphological studies of adult specimens, *M. katsurai* is classified into the subgenus *Chymophila* (Reemer and Stahls 2013). Our morphological studies on the *M. katsurai* puparium support this classification. There are several morphological similarities between the *M. katsurai* puparium and that of Microdon (Chymophila) fulgens, such as dorsal surfaces that have a uniform reticular structure, a thick marginal band consisting of both a dorsal fringe and a ventral fringe, and anterior spiracles with a height that is shorter than its width (Thompson 1981). Moreover, in the puparium of both species, a flat median carina divides the two areas of the opening of the spiracles at the spiracular plate of the posterior respiratory process, which is characteristic in the subgenus *Chymophila* (Thompson 1981). Considering all this together, the classification by puparium morphology in the present study is concordant with that of the adult morphology.

The two individuals that failed to emerge, No. 1 and No. 3, could not be identified using adults, but they are considered to be *M. katsurai* based on the morphology of their puparia.

In Japan, thus far, *M. katsurai* has been recorded in the Nagano, Ibaraki, Tochigi, Mie, Osaka, Hyogo, Yamaguchi, Kagawa and Kagoshima prefectures (Hironaga et al. 1998, Katsura 1998, Kano 1999, Tanaka 2002, Kusama and Tamaki 2004, Genka 2010, Iwai 2010, Sadahiro 2013, Noishiki 2014), and for the first time, it was collected in the Yamanashi prefecture in the present study. Although *M. katsurai* is often recorded in lowland environments (Maruyama and Hironaga 2004), it has also been found in habitats with relatively high altitudes of up to 600 m (Noishiki 2014). The altitude of the habitat where *M. katsurai* was found in the present study is approximately 600 m, suggesting that the habitat preference of this species is broader than previously speculated.

*M. katsurai* is a rare species that is designated as Vulnerable (VU) in Japan (Ministry of the Environment 2015). Furthermore, the habitat of *P. lamellidens*, which is designated as Vulnerable (VU) as well, is decreasing from the influence of recent residential and industrial development (Maruyama and Hironaga 2004, Maruyama et al. 2013, Sadahiro 2013, Ministry of the Environment 2015). The present study indicated a strong ecological association between *M. katsurai* and *P. lamellidens*. It is necessary to protect the *P. lamellidens* habitats immediately to conserve not only *P. lamellidens* but also the very rare species *M. katsurai*.

## Figures and Tables

**Table 1.** Days observed for each larva to reach pupal and adult stages after collection of the specimens from their habitat.

Individuals	Pupation	Appearance of anterior spiracle	Emergence of adult
No. 1	16	24	41
No. 2	24	27	48
No. 3	16	24	41
